# Low engagement in HIV services and progress through the treatment cascade among key populations living with HIV in Mozambique: alarming gaps in knowledge of status

**DOI:** 10.1186/s12889-020-10039-2

**Published:** 2021-01-15

**Authors:** Makini A. S. Boothe, Isabel Sathane, Cynthia Semá Baltazar, Noela Chicuecue, Roberta Horth, Erika Fazito, Henry F. Raymond

**Affiliations:** 1 Institute for Global Health Sciences, University of California (UCSF), San Francisco, USA; 2 Ghent University, Faculty of Medicine and Health Sciences, Ghent, Belgium; 3 The National Program of the Control of STIs and HIV/AIDS, Ministry of Health, Maputo, Mozambique; 4 National Institute of Health, Ministry of Health, Maputo, Mozambique; 5 ICAP, Columbia University, Pretoria, South Africa; 6 School of Public Health, Rutgers University, New Brunswick, USA

**Keywords:** Female sex workers, Key populations, HIV treatment cascade, Men who have sex with men, Mozambique, People who inject drugs

## Abstract

**Background:**

Mozambique has a generalized HIV epidemic of 13.5% among the general population. Early modeling exercises in Mozambique estimate that key populations (KP), defined as men who have sex with men (MSM), female sex workers (FSW), and people who inject drugs (PWID), along with their partners account for about one third of all new infections. There is limited data describing the engagement of KP living with HIV in testing, care and treatment services.

**Methods:**

We conducted a secondary data analysis of HIV-positive participants in the first Bio-behavioral Surveillance (BBS) surveys in Mozambique conducted 2011–2014 in order to assess service uptake and progress though the HIV treatment cascade among MSM, FSW, and PWID. Unweighted pooled estimates were calculated for each key population group.

**Results:**

Among HIV-positive MSM, 63.2% of participants had ever received an HIV test, 8.8% were aware of their status, 6.1% reported having been linked to care, while 3.5% initiated ART and were currently on treatment. Of the HIV-infected FSW participants, 76.5% reported a previous HIV test and 22.4% were previously aware of their status. Linkage to care was reported by 20.1%, while 12.7% reported having initiated ART and 11.8% reported being on treatment at the time of the survey. Among HIV-infected PWID participants, 79.9% had previously received an HIV test, 63.2% were aware of their HIV status, and 49.0% reported being linked to care for their HIV infection. ART initiation was reported by 42.7% of participants, while 29.4% were on ART at the time of the survey.

**Conclusion:**

Among the three high risk populations in Mozambique, losses occurred throughout critical areas of service uptake with the most alarming breakpoint occurring at knowledge of HIV status. Special attention should be given to increasing HIV testing and linkage to ART treatment. Future surveys will provide the opportunity to monitor improvements across the cascade in line with global targets and should include viral load testing to guarantee a more complete picture of the treatment cascade.

## Background

Key populations (KP), defined as men who have sex with men (MSM), sex workers and their clients, people who inject drugs (PWID), people in prisons and other closed settings, and transgender people are population groups disproportionally infected with HIV relative to their size due to their high-risk sexual and drug use behaviors [1, 2]. Their risk of infection is also enhanced by legal and social environments characterized by inadequate health services, stigma and discrimination, and human rights violations, all contributing to low engagement with HIV prevention, care and treatment services [1]. The HIV epidemic among KP has potentially far reaching effects at the population level and the World Health Organization describes the “multiplier effect,” whereby KP can transmit HIV to their sexual partners in the general population, such as female sexual partners of MSM, clients of FSW, and non-injecting sexual partners of PWID [1]. In addition, HIV-infected pregnant and breast-feeding women, who may themselves be KP or sexual partners of KP, are at risk of transmitting the infection on to their children [3]. For this reason, it is imperative to address HIV among KP given the impact of the disease on their health status, but also the potential impact on the health of the general population.

Mozambique has a generalized HIV epidemic, with a prevalence in the general population of 13.5% [4]. In Mozambique, KP are defined as MSM, female sex workers (FSW), and PWID. Early modeling exercises have estimated that KP and their partners account for about one third of all new infections in Mozambique [5]. The HIV National Strategic Plans called for special surveys to be conducted among these three population groups to estimate HIV prevalence, assess risk factors for HIV infection and estimate population size of these key populations [6, 7]. In response, the first round of bio-behavioral surveillance surveys (BBS) were conducted between 2011 and 2014 in three urban areas in Mozambique. To date, these surveys represent the only available surveillance data about KP in Mozambique.

In 2015, UNAIDS launched the 2020 Fast Track Targets by which to monitor the response to and progression of the HIV epidemic. The “90–90-90 targets” advocate for tracking key indicators along the treatment cascade with the goal of ending the AIDS epidemic by 2030 [8]. These ambitious targets aim to ensure that by 2020, 90% of people living with HIV (PLHIV) are aware of their status, of which 90% are on treatment (81% of the total), and finally 90% of them are virally suppressed (73% of the total). These targets provide an important benchmark by which to monitor Mozambique’s contribution to the global benchmark.

In 2015, the second National AIDS Indicator Survey (AIS), a population-based household survey that measures key HIV indicators, was conducted in Mozambique and provided the first nationally representative data about the engagement of the general population across the HIV treatment cascade [4, 9]. It is estimated that 40% of PLHIV are aware of their status, 35% are on treatment and 23% are virally suppressed; disaggregations of this cascade by sex highlight that men are less engaged than women at every step. These estimates are well below global targets, serve as a baseline for the general population. They also imply a worse situation for KP given the evidence that social, legal and structural barriers to prevention, care and treatment services severely limit the progress of KP through the cascade when compared to the general population [10].

In this context, the purpose of this secondary analysis of results from the BBS surveys is to examine available data from the first BBS surveys for baseline estimates describing the engagement of MSM, FSW, and PWID living with HIV in HIV testing, care and treatment services. The results provide important information for monitoring critical points along the cascade.

## Methods

### Survey design

The first round of BBS surveys among KP in Mozambique were conducted from 2011 to 2014 in an urban center from each of the three regions of the country – Maputo (MSM, FSW, PWID) in the south, Beira (MSM, FSW) in the center, Nampula (MSM, FSW) and Nampula/Nacala (PWID) in the north. Given the difficulty with reaching hard-to-reach populations, the probability-based peer-to-peer chain-referral recruitment methodology, respondent-driven sampling (RDS), was implemented [11, 12]. This methodology uses the social network size to produce weights, whereby adjusted estimates are representative of the target population in the geographical location where the survey is conducted. A comprehensive description of the survey study design for each survey has been previously published [13–18].

### Study population

Eligibility criteria for participants in the MSM survey included biological male sex, minimum age of 18 years old, reported having engaged in oral or anal sex with another male in the 12 months preceding the survey. For participants in the FSW survey, eligibility criteria included biological female sex, minimum age of 15 years old, and having received money in exchange for sex from someone other than a steady partner in the 6 months preceding the survey. Finally, for the PWID survey, participants were required to be at least 18 years of age and there was no restriction related to sex. Prior to December 2013, eligibility was based upon having reported injection drug use without a prescription in the 12 months preceding the survey. However, in an effort to account for slow recruitment patterns, this criteria was later adjusted to include any person who reported having ever injected drugs without a prescription.

For all three surveys, eligible participants were required to have lived, worked or socialized in the survey city at least during the 6 months preceding the survey and they must have been in possession of a valid referral coupon received from a peer; anyone reporting previous participation in the study was excluded. Written informed consent was required separately for both the behavioral questionnaire and biological testing; enrollment in the study was contingent upon providing consent to the behavioral questionnaire. For the purpose of the present analysis, participants who did not respond to at least 90% of the behavioral questionnaire and/or did not consent to have an HIV test result were excluded (*n* = 53 MSM, *n* = 6 FSW, *n* = 47 PWID).

Recruitment period was from July to November 2011 (MSM), September 2011 to March 2012 (FSW) and October 2013 to March 2014 (PWID).

### Study measures

The number of PLHIV was computed based on confirmatory central-level ELISA test results (MSM, FSW) or by combining rapid test results for new-positives with self-report of known-positives (PWID). The complete questionnaires for the three have been previously [13–15]. For the purpose of this analysis, HIV testing uptake was assessed based on the question, “*Have you been tested for HIV?*” Knowledge of seropositive status was determined based on responses to the question, “*What do you think is your HIV status today?*” Linkage to care was assessed based on responses to the question, “*Have you seen a nurse, doctor or other health care provider for a medical evaluation or care related to your HIV infection?*” (MSM) or “*Have you sought a doctor, nurse, or other health care professional for medical examinations or treatment for your HIV infection?*” (FSW, PWID). ART status was assessed by the question, “*Have you ever taken or are you currently taking ARTs? (Antiretrovirals are medicines that slow the growth of the virus in HIV-infected people and enable people with AIDS to live for a much longer time),*” whereby ART initiation and current on treatment were coded separately.

### Statistical analysis

Unweighted pooled estimates were calculated for each area of service uptake due to the low sample size of key populations in each survey city. There are various approaches to constructing an HIV cascade depending on the desired outcome of interest when assessing progress toward the Fast Track targets [10, 19, 20]. For the purpose of this analysis, the total number of PLHIV was the denominator of each step along the HIV treatment cascade; viral load suppression was not assessed. This approach provides insight into infection and potential HIV transmission. Data analysis was conducted using SAS version 9.4 (SAS Institute, Cary, NC, USA).

## Results

### Recruitment

**Fig. 1:**
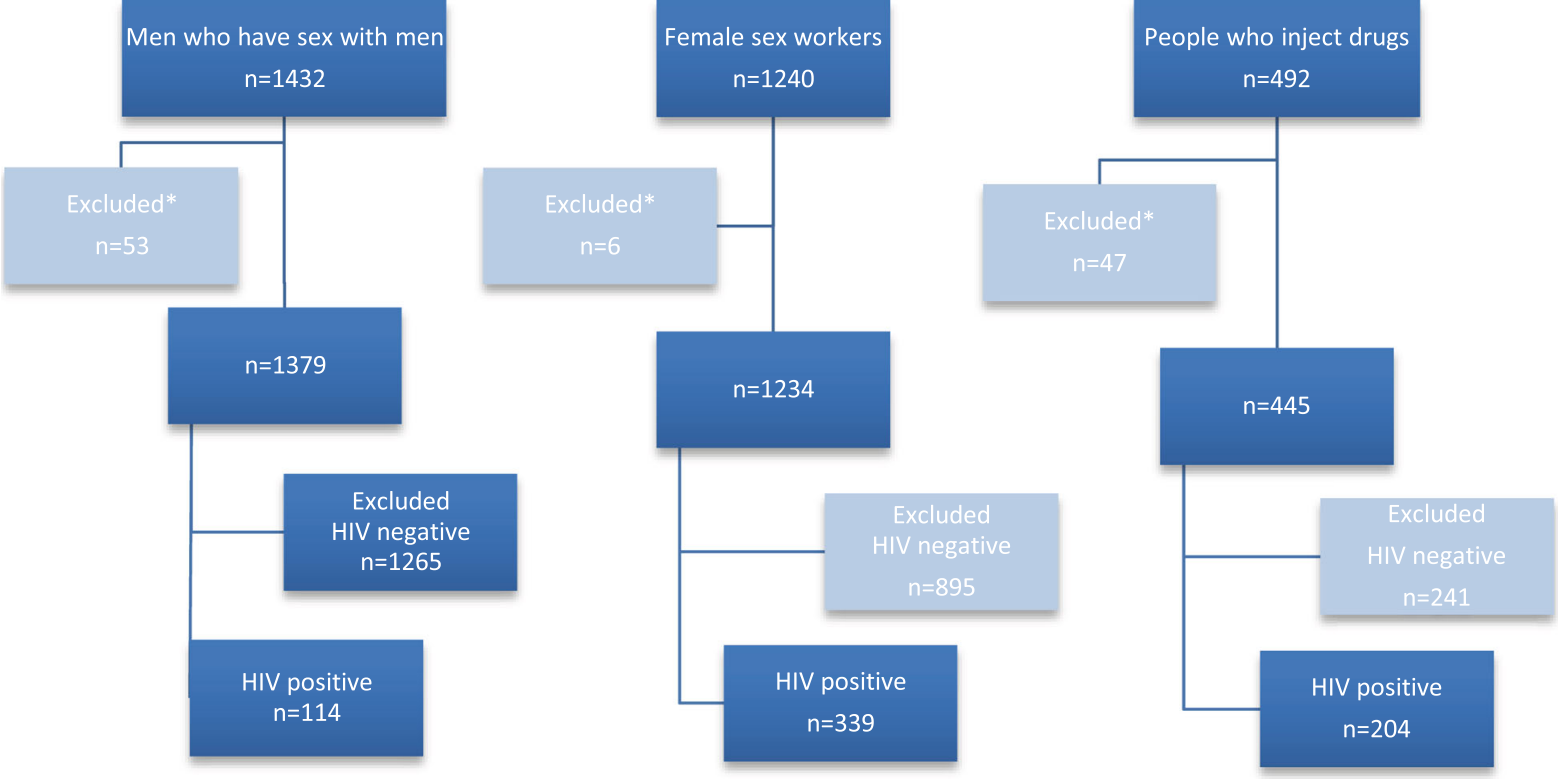
BBS Recruitment and Analysis Flow, Mozambique 2011–2014. * Individuals who did not respond to at least 90% of the behavioral questionnaire and/or did not consent to have an HIV test result were excluded from analysis

Figure 1 outlines the recruitment and analysis flow of the surveys where 1379 MSM (447 Maputo, 581 Beira, 351 Nampula), 1234 FSW (397 Maputo, 409 Beira, 428 Nampula), and 445 PWID (319 Maputo, 126 Nampula/Nacala) had an HIV test result. Among those, 8.3% of MSM (*n* = 114), 27.5% of FSW (*n* = 339) and 45.8% of PWID (*n* = 204) were positive for HIV and were included in the final analysis.

### Engagement in care

Table 1 outlines the engagement of HIV-positive MSM, FSW and PWID participants in testing, care and treatment services. Of the 114 MSM who tested positive for HIV (*n* = 50 Maputo, *n* = 53 Beira, *n* = 11 Nampula), 63.2% (*n* = 72) reported ever receiving an HIV test, 8.8% (*n* = 10) were aware of their status, 6.1% (*n* = 7) reported having been linked care, while 3.5% (*n* = 4) initiated ARTs and were currently on treatment; thus 42.9% were not retained in treatment.

**Table 1:** Engagement in HIV Services among MSM, FSW and PWID in Mozambique, 2011–2014

	MSM (*N* = 114)		FSW (*N* = 339)		PWID (*N* = 204)	
Services	n	%	n	%	n	%
Previous HIV Test	72	63.2%	261	77.0%	163	79.9%
Knowledge of HIV status	10	8.8%	76	22.4%	129	63.2%
Linkage to HIV services	7	6.1%	70	20.6%	100	49.0%
Initiated ART Treatment	4	3.5%	43	12.7%	87	42.6%
Currently on ART Treatment	4	3.5%	40	11.8%	60	29.4%

Of the HIV-infected FSW participants (*n* = 339), 76.5% (*n* = 261) reported a previous HIV test and 22.4% (*n* = 76) were aware of their status at the time of the survey. Linkage to care was reported by 20.1% (*n* = 68), while 12.7% (*n* = 43) reported having initiated ART and 11.8% (*n* = 40) reported being on treatment at the time of the survey; thus 93.0% of FSW who initiated treatment were retained in care at the time of the survey.

Among HIV-infected PWID participants (*n* = 204), 79.9% (*n* = 163) had previously been tested for HIV, 63.2% (*n* = 129) were aware of their HIV status and 49.0% (*n* = 100) reported being linked to care for their HIV infection. ART initiation was reported by 42.7% of participants (*n* = 87), while 29.4% (*n* = 60) were on ART at the time of the survey; thus there was 69.0% treatment retention among PWID participants.

### Progress through the HIV treatment Cascade

**Fig. 2:**
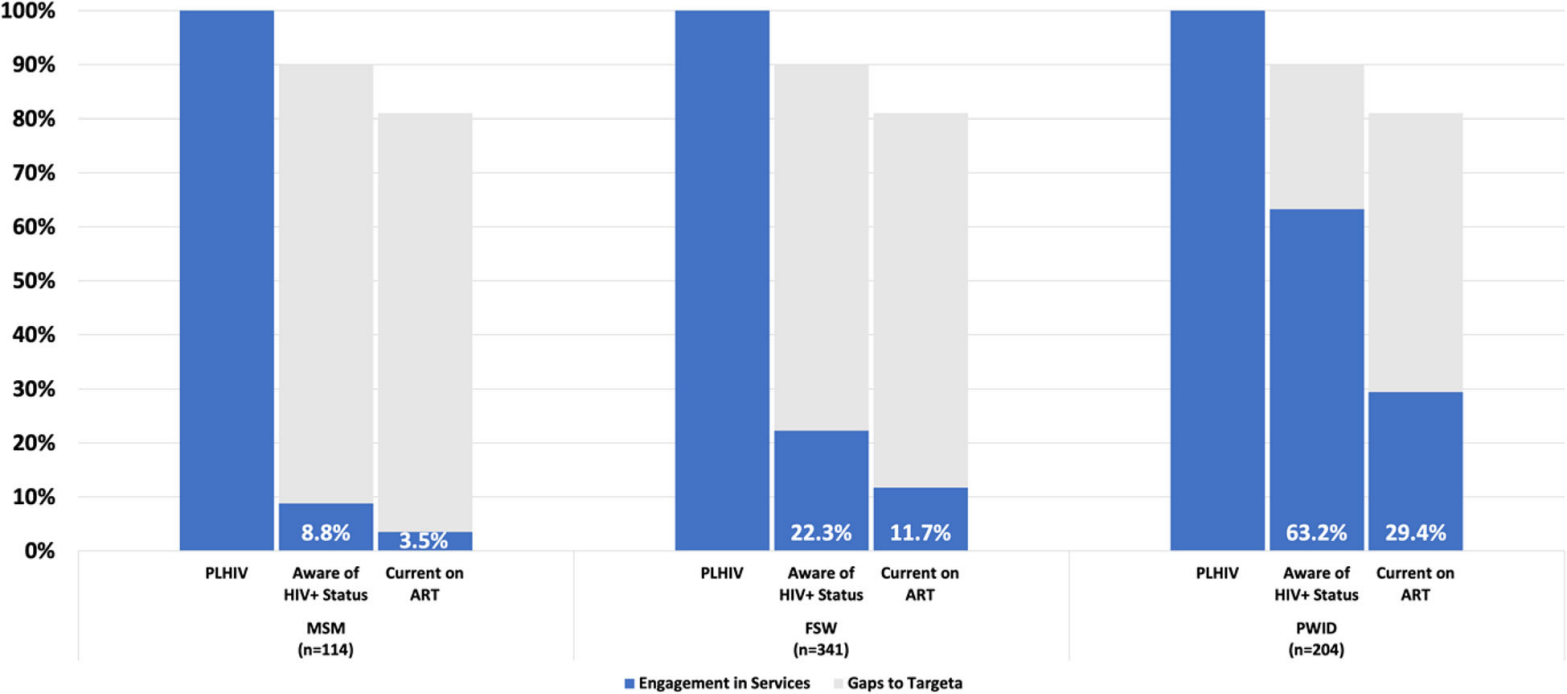
HIV Treatment Cascade among MSM, FSW and PWID in Mozambique, 2011–2014. PLHIV: People Living with HIV. Gaps in Target: The baseline shortfall between service engagement and the achievement of the Fast Track Targets; viral suppression data not available

Figure 2 displays the unadjusted pooled estimates for the number of participants within each key population group as they advance through the HIV treatment cascade. Participants in all three groups fell well below global Fast Track Targets for knowledge of HIV status: 8.8% (MSM), 22.4% (FSW), 63.2% (PWID) and currently on treatment: 3.5% (MSM), 11.8% (FSW), 29.4% (PWID); viral load data was not available.

## Discussion

Gaps were identified throughout the HIV treatment cascade for the three populations, with the largest breakpoint occurring at knowledge of HIV status; all key populations fell below the 90% target for every indicator although this was most stark among MSM where only 8.8% had knowledge of their HIV status prior to the survey. Furthermore, results show that of the MSM, FSW and PWID participants who were aware of their status, only 40.0, 52.6, and 47.2%, respectively reported being on treatment at the time of the survey. This translates to two in five HIV-positive MSM participants, and one in two for both HIV-infected FSW and PWID participants. However, once initiated on ART, both MSM and FSW were retained above the 90% target, while a laudable achievement this only represents a small fraction of the HIV-positive population. In general terms, use of services appears higher among PWID, when compared to the other KP groups; however, PWID are still well below global targets.

When compared to other KP groups in the region, HIV testing uptake among survey participants was consistent with previous studies [21, 22]. However, MSM and FSW surveys in Mozambique reported lower awareness of their HIV status compared to those same groups in other Sub-Saharan African countries, although PWID participants reported higher awareness than other PWID in the region [23–28]. ART engagement was also lower across all populations groups [23, 27, 29, 30]. In the few studies with results of viral load suppression in Sub-Saharan Africa, there was a range of 11%-42 among MSM, 11.0–49.5% among FSW and less than 5% in among PWID in Kenya [24–28]. The differences in results may be attributed to different survey measures, recruitment methodology, policies and interventions. However, accurate and quality data examining the cascade in Sub-Saharan Africa across the three population groups is still scarce ( [27]. Nevertheless, to our knowledge, no countries in Sub-Saharan Africa have been able to meet ambitious 90–90-90 Fast Track Targets for key populations.

Mozambique’s most recent AIDS Indicator Survey, a nationally representative household survey, was conducted in 2015 and included indicators to monitor service uptake and the treatment cascade within the general population [4, 9]. History of HIV testing among people living with HIV (PLHIV) in the general population compared to HIV-infected key populations was similar among females and lower among men: 73.6% (females) and 55.8% (males) compared to 63.2% (MSM), 76.5% (FSW) and 79.9% (PWID). PLHIV in the general population were much more aware of their status than HIV-infected KP: 46.2% (females) and 28.1% (males) compared to 8.8% (MSM), 22.3% (FSW) and 63.2% (PWID), demonstrating major programmatic challenges to linking these vulnerable populations to care and treatment services. Although ART initiation was not assessed in the AIS, all three KP reported lower current enrollment in ART treatment than the general population: 40.0% (females) and 23.0% (males) compared to 3.5% (MSM), 11.8% (FSW), and 29.4% (PWID), however, the data highlights the pressing need for improved HIV treatment services for all population groups. The results do not include viral load estimates for KP, however given that viral suppression among PLHIV in the general population are greatly below global targets at 36.9% (females) and 22.4% (males), we can assume that the outcomes among KP are considerably lower given their lower engagement in services compared to the general population as well as the evidence of social, legal and structural barriers to access health care services [10]. The low evidence of engagement in health services among MSM is consistent with low health service access among men in the general population in Mozambique [4].

Finally, the results must be understood within the context of the care and treatment landscape when the surveys were implemented. Mozambique’s National Acceleration Plan was introduced in 2013, which changed treatment guidelines and vastly scaled up the availability of ART in the country. The roll-out of increased ART provisions occurred after the BBS was implemented among MSM & FSW. This change in the landscape may explain why PWID reported greater engagement in the care compared to the other KP groups. Despite this policy change however, there is still evidence in the most recent AIDS Indicator Survey of low engagement of the general population in the HIV treatment cascade, where men are less engaged than women, and thus it can be posited that even with greater access to ART services, social and structural barriers continue to impact KP engagement in health services.

More recent data estimate that in 2019, 77% of PLHIV are aware of their status, 60% were on treatment and 45% are virally suppressed, however viral load testing was only available for about half of PLHIV on treatment and therefore must be interpreted with caution. Viral load testing is not yet universally available due to insufficient patient/provider demand and systemic issues such as inadequate information flow and limited technology [31]. However, the next round of BBS surveys will provide updated estimates of KP engagement in the HIV testing and treatment cascade and will include viral suppression estimates that can provide a point of comparison with those of the general population.

This is the first analysis of engagement in HIV services and progress through the HIV treatment cascade among key populations in Mozambique. Although this analysis offers important insights into the use of HIV treatment services among key populations in Mozambique, it is important to acknowledge this analysis’ limitations.

While RDS is a robust methodology for sampling among hidden populations, there are some inherent limitations such as selection bias in chain referral sampling methods, recall bias and social desirability bias for self-reported risk behaviors; non-response bias also applies to individuals who did not consent to an HIV test – and were subsequently removed from the analysis –however the proportion was relatively low (3.70% MSM, 0.24% FSW, and 9.55% PWID). PWID population definition was modified in the middle of recruitment due to slow recruitment patterns, from injection in the last 12 months to ever having injected drugs without a prescription. Only 2% of the sample across both PWID survey cities reported not injecting in the past 12 months and therefore this change in recruitment likely did not have a large impact on the overall size estimation. Nevertheless, the change of definition may have an impact on the comparability of the results to other populations and future BBS surveys. Additionally, some respondents may have been previously aware of their HIV positive status but refused to disclose due to stigma or social desirability given the common knowledge that HIV-positive individuals should be engaged in care and treatment. This potential selective underreporting of HIV status has been observed in previous studies, and can underestimate prevalence results and subsequent cascade assessments [24, 25, 32, 33]. As an illustrative example, in the most recent AIS conducted in Mozambique, only 26.1% of PLHIV self-reported current ART use however, when biomarker testing was used, the number of current on ART increased to 35% indicating that individuals did not self-disclose their status [4].

Next, the computation of unweighted pooled estimates among survey participants means that results are not generalizable to the KP in the geographic locations where the surveys were conducted nor to other cities in Mozambique, but instead only represent survey participants. Due to the low sample size, it was not possible to conduct meaningful weighted estimates of the cascade for the KP in each survey city. However, the results provide the best available proxy indication of engagement in services and progress through the HIV treatment cascade and highlight the need for enhanced efforts targeted to these groups.

Finally, the data was collected in 2011–2014 and may not represent the current situation of KP in the country, however, they remain the only available surveillance data about KP Mozambique and the best available indication of engagement in services and progress through the HIV treatment cascade. The continued absence of more updated surveillance data limits the comprehensive monitoring of the HIV epidemic and demonstrates the need to strengthen the HIV surveillance system in the country.

## Conclusions

The results of this analysis highlight the necessity for interventions that increase uptake of HIV testing and ART treatment among all KP, but particularly among MSM and FSW who reported less engagement than PWID. Since the implementation of the first round of BBS surveys among KP in Mozambique, National Guidelines were adopted outlining a standard package of 10 services for KP such as HIV counseling and testing; STI screening; and provision of condoms and lubricants [34]. Additionally, in 2017, Mozambique began the roll-out of the test and start strategy with specific emphasis on individuals belonging to key population groups.

Follow-up BBS surveys will provide important data points to assess trends and monitor improvements in KP engagement in the health system as a result of these policies. In addition, the inclusion of viral load testing in future surveys will guarantee a more complete picture of the HIV testing and treatment cascade among populations most vulnerable to HIV transmission. In the meantime, the results from this analysis can be applied to size estimation exercises to support target setting and resource allocation. Modeling exercises can also use these and programmatic data to assess the burden of the HIV epidemic attributable to key populations in Mozambique. Finally, improved health information systems at the community and facility level for KP can also provide more up to date information on their progression through the cascade.

## Data Availability

The dataset analysed for the current study are fully available from the Data Management Unit of the Mozambique National Institute of Health (INS) data repository for researchers who meet the criteria for access to confidential data following the submission of a concept note. For information, please visit: www.ins.gov.mz or contact: secretaria@ins.gov.mz.

## Abbreviations

AIS: AIDS Indicator Survey
ART: Antiretroviral
BBS: Biological and behavioral Survey
KP: Key populations
FSW: Female sex workers
HIV: Human immunodeficiency virus
MSM: Men who have sex with men
PLHIV: People living with HIV
PWID: People who inject drugs
RDS: Respondent-driven sampling
STI: Sexually transmitted Infection
WHO: World Health Organization
